# New Insights into the Composition of Aggregation Pheromones in *Polygraphus poligraphus*, *Polygraphus punctifrons*, *Polygraphus subopacus* and *Polygraphus proximus*

**DOI:** 10.1007/s10886-025-01577-3

**Published:** 2025-02-07

**Authors:** Lina Viklund, Joakim Bång, Martin Schroeder, Erik Hedenström

**Affiliations:** 1 Eco-Chemistry, Department of Natural Science, Design and Sustainable Development, Mid Sweden University, 851 70 Sundsvall, Sweden; 2 Department of Ecology, Swedish University of Agricultural Sciences, Box 7044, 750 07 Uppsala, Sweden

**Keywords:** Bark beetles, SPME, EAG, GC–MS/EAD using chiral column, Field studies, Interactions

## Abstract

Four-eyed bark beetles of the genus *Polygraphus* have been involved in large bark beetle outbreaks in different parts of the world, resulting in major economic losses. A striking example is the invasive species *Polygraphus proximus* which is a pest on *Abies sibirica* in Russia. In Sweden, *Polygraphus poligraphus* has been involved in bark beetle outbreaks on Norway spruce, *Picea abies*, together with the European spruce bark beetle *Ips typographus*. Two related species, *Polygraphus punctifrons* and *Polygraphus subopacus* are also present in Sweden. Recently, aggregation pheromones or pheromone components have been identified for these four *Polygraphus* species. However, questions remain regarding the complete composition of their pheromones, particularly for *P. subopacus* and *P. proximus,* whose aggregation pheromones appear to be very similar. In an attempt to better understand the chemical communication of these species, additional studies were conducted on *P. poligraphus*, *P. punctifrons* and *P. subopacus* using solid phase microextraction coupled with gas chromatography and mass spectrometry (SPME–GC–MS), electroantennography (EAG) as well as SPME–GC–MS and GC–MS with electroantennographic detection (EAD). Field experiments were also conducted. In *P. punctifrons*, some male-specific compounds were found in addition to those previously identified. In EAG and SPME–GC–MS/EAD studies, all three *Polygraphus* species responded strongly to grandisol. Using a chiral column, GC–MS/EAD revealed that they were able to detect both enantiomers of grandisol. In summary, this work presents our current understanding of the aggregation pheromones in four *Polygraphus* species and the challenges we have met in identifying species-specific pheromone blends for some of these species.

**Supplementary Information:**

The online version contains supplementary material available at 10.1007/s10886-025-01577-3.

## Introduction

Bark beetles are part of the weevil family (Coleoptera: Curculionidae) and belong to the subfamily Scolytinae. Of more than 6000 species worldwide, only a minority are able to attack and kill healthy trees. However, these species can have a profound economic and environmental impact (Raffa et al. 2015). In Europe, an average of 23 million m^3^ of timber was damaged annually by bark beetles between 2010–2019, reaching 70 million m^3^ in 2019 and the trend kept increasing during 2020 and 2021 (Patacca et al. 2022). The impact of bark beetle outbreaks is expected to continue increasing due to climate change with annual damage in central Europe projected to be nearly six times higher in 2021–2030 as compared to 1971–2010 (Hlásny et al. 2021). Worldwide, some of the most economically important species of bark beetles are the mountain pine beetle, *Dendroctonus ponderosae*, in North America, the European spruce bark beetle, *Ips typographus*, in Europe (Grégoire et al. 2015) and the four-eyed fir bark beetle, *P. proximus*, in Russia (Krivets et al. 2019). Secondary pest species can also be economically important, such as *Polygraphus rufipennis* in North America which can kill trees weakened by other pests, and speed up the deterioration of wood in dying trees (Bowers et al. 1996).

The largest bark beetle outbreak ever documented in Sweden started in 2018 after an exceptionally warm and dry summer. Approximately 33 million m^3^ of Norway spruce (*Picea abies*) was killed in southern Sweden between 2018–2023 (Schroeder and Kärvemo 2022; Wulff and Roberge 2023). The European spruce bark beetle, *I. typographus*, was the dominating species in this outbreak, but *P. poligraphus* was also commonly found under the bark of killed trees (Schroeder 2023; Wulff and Roberge 2023). In previous bark beetle outbreaks in central Sweden, *Polygraphus* species (mainly *P. poligraphus*) have caused an unexpected amount of damage, but their role in bark beetle outbreaks is not completely understood (Wulff and Hansson 2013). There are three species of *Polygraphus* in this part of Sweden; *P. poligraphus*, *P. punctifrons* and *P. subopacus.* The flight of these three species generally starts after the onset of flight of *I. typographus*, and peaks during July and August (Ehnström and Axelsson 2002; Viklund et al., unpublished results). The invasive four-eyed fir bark beetle *P. proximus*, which has caused extensive damage to forests of *Abies sibirica* in Russia, is likely to spread to Sweden and the rest of the European Union in the near future, where it threatens to attack species of the genera *Abies, Pinus, Picea, Larix* and *Tsuga* (EPPO 2014; De la Peña et al. 2020).

Tree-killing bark beetles use aggregation pheromones in order to communicate with each other and to initiate mass attacks on their host trees. Pheromone production in the pioneering sex is usually induced by feeding and the aggregation pheromone is attractive to both sexes. Sometimes both sexes can contribute to pheromone production. Pheromones can be produced *de novo* by the beetles, by derivatization of host tree precursors or possibly by beetle-associated bacteria or fungi (Blomquist et al. 2010). Once aggregation pheromones are identified and synthesized, they may be used as baits in traps in order to monitor beetle populations and investigate flight activity patterns, and to manage infestations by mass trapping or baiting of trap trees (Fettig and Hilszczański 2015).

*P. poligraphus*, *P. punctifrons* and *P. subopacus* are polygamous species and the male initiates the colonisation of host trees by creating a nuptial chamber in the bark and by producing aggregation pheromones. Males are the pioneering sex also in *P. proximus* although it is a monogamous species (Kerchev 2014). Male-specific compounds that are part of the aggregation pheromones have been identified in these four species. The aggregation pheromone of *P. poligraphus*, (*–*)-(*R*)-terpinen-4-ol (Schurig et al. 1985), is emitted by males in high enantiomeric purity (> 96.3% ee) which decreases after mating to 67.7% ee (Rahmani et al. 2015). The (+)-(*S*)-enantiomer acts as a repellant for the beetles. When the enantiomeric purity of (*–*)-(*R*)-terpinen-4-ol is 50% ee or less, it is no longer attractive unless it is combined with racemic frontalin, even though frontalin is not emitted by *P. poligraphus* (Viklund et al. 2019; Rahmani et al. 2015). *P. punctifrons* males produce ( +)-(*1R,2S*)-grandisol and (*–*)-(*R*)-terpinen-4-ol, but both sexes can be efficiently caught in traps baited with racemic grandisol. If (*–*)-(*R*)-terpinen-4-ol is combined with *rac*-grandisol, catches of *P. punctifrons* increase and *P. poligraphus* is also caught in the traps (Rahmani et al. 2019). In *P. subopacus,* several male-specific compounds have been found but so far, only (*Z*)−2-(3,3-dimethylcyclohexylidene)-ethanol [(*Z*)-DMCHE] has been proven attractive. Baiting traps with this compound alone results in by-catches of *P. poligraphus* (Viklund et al. 2021). *P. proximus* males appear to produce a nearly identical blend of compounds as *P. subopacus* males, where (*Z*)-DMCHE and 3-methyl-2-buten-1-ol are attractive to the beetles (Viklund et al. 2022). If (*Z*)-DMCHE is used as bait for *P. proximus,* with or without the addition of 3-methyl-2-buten-1-ol, *P. subopacus* is also caught in the traps, indicating that this is not a complete aggregation pheromone. Thus, efficient species-specific pheromone baits for *P. subopacus* and *P. proximus* have not yet been developed.

When bark beetle pheromones were first investigated, the pheromone of a species was often thought to consist of a single compound. More recently, it is clear that most bark beetle pheromones consist of two or more compounds and that single component pheromones are very rare (Birgersson et al. 2012; Tillman et al. 1999; Silverstein & Young 1976). Aggregation pheromone components often overlap significantly among species, genera and tribes (Raffa et al. 2015). Species-specificity and reproductive isolation may be achieved by differences in semiochemical blends, enantiomeric composition and ratios of pheromone components as well as host species fidelity, niche-separation within the same host, temporal and geographic isolation or behavioral and physiological incompatibility. Responses to pheromones and host tree volatiles within a species may vary during the season and between different geographic locations (Raffa et al. 2015). Bark beetles can also communicate by acoustic signals and species-specific differences have been found in *Polygraphus* species (Kerchev 2020).

In addition to pheromones, bark beetles are often attracted to host tree volatiles and compounds which indicate stress, decay or microbial infections in their host trees (Raffa et al. 2015). Most bark beetles seem to be closely associated with symbiotic fungi which may help the beetles overcome tree defences by metabolizing toxins, or may serve as a source of nutrition (Raffa et al. 2015; Kandasamy et al. 2016). Volatiles from symbiotic fungi can function as synergists of bark beetle attractants, whereas other fungal volatiles can synergize the effects of repellant or anti-attractant compounds (Kandasamy et al. 2016). Anti-aggregation pheromones or repellants, which can be produced by the beetles, by non-hosts or by beetle-associated microorganisms as they degrade host tree materials, can be used to protect individual trees or forest stands. They can also be used in push–pull strategies where repellants are combined with attractants in order to divert beetles from high-value stands to traps or trap trees (Fettig and Hilszczański 2015).

Because *Polygraphus* species in Sweden and Russia share pheromone components, have overlapping flight periods (Viklund et al. 2022; Viklund et al., unpublished results), are partially sympatric, and share some host species, it is likely a species-specific pheromone blend exists for each species. Solid-phase microextraction has previously been used together with gas chromatography and mass spectrometry (SPME–GC–MS) to identify sex-specific compounds emitted from *P. poligraphus, P. punctifrons, P. subopacus* and *P. proximus* (Rahmani et al. 2015, 2019; Viklund et al. 2021, 2022). However, in *P. poligraphus* and *P. punctifrons* only a few emitted compounds have been identified and it seems likely that these do not constitute the complete aggregation pheromones of these species. As our methods and selection of GC-columns have been refined over the years, we decided to conduct some additional studies on these two species to try to identify additional sex-specific compounds. For *P. subopacus* and *P. punctifrons*, we decided to use SPME–GC–MS with electroantennographic detection (EAD) to further investigate the composition of their pheromones and to search for compounds which *P. poligraphus* could detect and possibly be repelled by. Grandisol has previously been identified as a male-specific volatile in both *P. punctifrons* and *P. subopacus* and is a pheromone component in other species of weevils (Bandeira et al. 2021). As there are two enantiomers of grandisol, we also used GC–MS/EAD with a chiral column to determine whether the antennae from each *Polygraphus* species could detect both enantiomers or just one, and whether there were any quantitative differences in their responses. Electroantennography (EAG) has previously been used to identify antennally active sex-specific compounds in *P. poligraphus* and *P. subopacus* (Viklund et al. 2019, 2022). Additional EAG studies, mainly with *P. punctifrons,* were conducted as a complement to the other studies and the results could be used to guide us in compound selection for field experiments.

In summary, we herein present our current knowledge of the chemical composition of the aggregation pheromones of the four aforementioned *Polygraphu*s bark beetles, as determined by SPME–GC–MS studies, EAG studies, studies using SPME–GC–MS/EAD, GC–MS/EAD with a chiral column and field studies. We also discuss the function of sex-specific compounds in the pheromones of each species.

## Methods and Materials

### Origin of Insects and Stem Sections Used in Laboratory Studies

Beetles were caught in the field in pheromone-baited traps which were emptied weekly. For *P. poligraphus*, traps were baited with 50 mg of (*–*)-(*R*)-terpinen-4-ol 50% ee and 50 mg of *rac*-frontalin dissolved in 8 mL of *n*-nonane and contained in a 12 mL glass vial with a drilled hole in the lid. The compounds were allowed to evaporate through a teflon tube (8 cm × 1.5 mm i.d.) lined with cotton yarn. *n*-Nonane was used as solvent to control the evaporation rate. The average evaporation rate measured in a fume hood over 15 days (22–25 °C, air flow 0.5–0.6 m/s) was 0.75 mg/day for each compound (Viklund et al. 2019). For *P. punctifrons*, wick-baits were constructed in the same way but loaded with 50 mg of *rac*-grandisol dissolved in 8 mL of *n*-nonane. Beetles were identified and sexed according to the methods described by Lekander (1959) using a stereomicroscope with 14*–*90 × magnification. Live beetles were kept in plastic jars with damp paper and holes in the lid in 8 °C for 1*–*7 days before they were used for experiments. Stem sections (diameter 10–15 cm, length 50–60 cm) were taken from felled Norway spruce (*Picea abies*) and were stored in a freezer (− 18 °C) for 1*–*3 months until they were used.

### SPME–GC–MS/EAD Studies of *P. punctifrons*, *P. poligraphus*, and *P. subopacus* Feeding on Stem Sections of Norway Spruce

In 2016 and 2018 we placed individual males and females of *P. poligraphus* on stem sections of Norway spruce (*Picea abies*). In 2021, we did the same thing but with *P. punctifrons*. Volatiles were collected and analyzed with SPME–GC–MS. Eppendorf tubes, with the bottom and lid cut off, were pinned to the stem sections. One beetle was introduced into each Eppendorf tube and the open end was covered with aluminium foil. There were 12 Eppendorfs pinned to each stem section and volatiles were sampled 3–7 days after the beetles had started boring into the bark. The most active individuals, determined by the amount of frass they produced, were chosen for SPME sampling. The spruce tree background was sampled from an empty Eppendorf tube where a hole (2.5 mm diameter) had been drilled manually in the bark. Boring insects were sampled for 1 h with a pink SPME fiber (65 μm polydimethylsiloxane/divinylbenzene, 57,293-U, Supelco, Bellefonte, PA, USA) which was introduced into the Eppendorf tube through the aluminium foil, and the collected volatiles were desorbed in the GC inlet for 5 min (250 °C, splitless injection, flow rate 1 ml/min with helium as the mobile phase). Compounds were separated on an HP5-MS column (30 m × 0.25 mm i.d., 0.25 μm film thickness; Agilent J&W Scientific, Folsom, CA, USA) with a temperature program starting at 50 °C for 2 min, then increasing by 10 °C per min up to 250 °C, where it was held for 5 min. The GC was a Hewlett-Packard 6890 N (Agilent Technologies, Santa Clara, CA, USA) equipped with an HP 5973 mass spectrometer (MS) operating in electron impact (EI 70 eV) ionisation mode. Transfer line was set to 250 °C. Raw MS data was analyzed using the Workstation 7.0.0 (Agilent) software. Compounds were initially identified by comparing their mass spectra with the NIST 14 library followed by final identification when comparing the mass spectra and the retention times with those of synthetic references (Viklund et al. 2021, 2022).

In 2021, males of *P. subopacus* and *P. punctifrons* were sampled with SPME as described above, but with a collection time of 30 min and the collections were now analyzed with GC–MS/EAD. The collected volatiles were tested for activity on the antennae of the same species and also on antennae of *P. poligraphus*, to identify compounds the latter species could detect and possibly be repelled by. The antenna responses were recorded with glass electrodes filled with Beadle-Ephrussi Ringer Solution (Ephrussi and Beadle 1936). The probe, micro manipulators, Probe Amp and CS-55 stimulus controller were all from Ockenfels SYNTECH GmbH, Buchenbach, Germany. The signal from the probe was connected through the Probe Amp to an Agilent 7890B GC (Agilent Technologies, Santa Clara, CA, USA) equipped with an Agilent 5977B mass spectrometer (MS). The MS was operating in electron impact (EI 70 eV) ionisation mode using helium as mobile phase (flow rate 1 mL/min), with the inlet set to 250 °C. The MS transfer line was set to 250 °C and the EAD transfer line to 200 °C. An HP-5MS column (30 m × 0.25 mm i.d., 0.25 μm film thickness; Agilent J&W Scientific, Folsom, CA, USA) was used, with a temperature program starting at 50 °C and increasing by 20 °C per min up to 250 °C. The collected volatiles on the SPME fiber were desorbed for 5 min in the inlet, with a split setting of 1:20. The raw data was analyzed using Agilent ChemStation software. The reference electrode was connected to the decapitated head of the insect and the recording electrode to the tip of one of the antennae. A constant flow of humified air (50 mL/min) was applied 1 cm above the antenna. 10 µl of a control substance (the main pheromone component of the species, diluted in *n*-hexane to 100 ng/μl) was applied to a piece of 00A grade filter paper (1 cm^2^) that was folded and inserted to a Pasteur pipette. The pipette tip was inserted into the stream of humified air from the stimulus controller and a short air puff (0.5 s) through the pipette delivered the substance to the antenna. This was done at the start and end of each run to monitor the condition of the antenna. There was a 3.5 s time lag between the MS signal (TIC) and the antenna response. The signals were edited in OpenChrom (Lablicate GmbH) where the time difference was adjusted and the signal from the antennae was inverted, making Fig. 3–7 easier to read and interpret.

### GC–MS/EAD Using a Chiral Column

Antennal responses were recorded in the same way as described above, but using a different GC column. A Beta DEX™ 225 chiral column (30 m × 0.25 mm i.d. and 0.25 μm film thickness, Supelco, Bellefonte, PA, USA) was used, with a temperature program starting at 50 °C and increasing by 2 °C per min up to 130 °C. We injected 1 µl of a standard solution of racemic grandisol (100 ng/µl) in splitless mode. Due to the long analysis time and the short lifespan of the antenna after decapitation, the head was mounted 8–10 min before the grandisol peaks eluted.

### EAG Studies of *P. poligraphus*, *P. punctifrons* and *P. subopacus*

Several compounds were tested for antennal response of *P. punctifrons* males and females (Table 1). We also determined responses of *P. poligraphus* and *P. subopacus* antennae to compounds that had not previously been tested by Viklund et al (2019, 2021). *P. proximus* is classified as a quarantine species in the European Union (De la Peña et al. 2020) and thus could not be brought into Sweden for EAG studies. Antennal responses were recorded with the same equipment as we used for our EAD studies. The compounds to be tested were diluted in *n*-hexane to concentrations of 100 ng/μl. 10 μl of each test solution was applied to a 00A grade filter paper (1 cm^2^) that was folded and inserted to a Pasteur pipette. The filter paper was dried for at least 5 min before the experiment started. The pipette was inserted to the stream of humified air from the stimulus controller and a short air puff through the pipette delivered the substance to the antenna. *n*-Hexane was used as a blank and (*Z*)-DMCHE, *rac*-grandisol or (*–*)-(*R*)-terpinen-4-ol were used as a control of the response of the antenna. All compounds could not be tested on all species with EAG, due to the availability of insects and compounds as well as difficulties in measuring antennal responses for some individuals, in particular for *P. punctifrons*.

### Chemicals

(*–*)-(*R*)-Terpinen-4-ol (50% ee) was purchased from TCI (Portland, OR, USA) and *rac*-frontalin was from Synergy Semiochemicals Corp (Burnaby, BC, Canada). (*Z*)- and (*E*)−2-(3,3-dimethylcyclohexylidene)-ethanol [(*Z*)- and (*E*)-DMCHE; Grandlure II and (*E*)-isomer of Grandlure II], racemic grandisol (Grandlure I) and a 1:1 mixture of (*Z*)- and (*E*)-2-(3,3-dimethylcyclohexylidene)-acetaldehyde [(*Z*)-and (*E*)-DMCHA; Grandlure III and IV] were from Bedoukian Research (Danbury, CT, USA). Geraniol was bought from Acros Organics (Geel, Belgium) whereas benzaldehyde, benzyl alcohol and 2-phenylethanol were purchased from Sigma-Aldrich (Schnelldorf, Germany). *n*-Nonane 99% was bought from Alfa Aesar (Heysham, Lancashire, UK). (*–*)-(*R*)-Terpinen-4-ol (99% ee) was purified by recrystallization of its 3,5-dinitrobenzoyl derivative, according to a method described previously (Viklund et al 2019). (*Z*)- and (*E*)-DMCHA were separated by flash chromatography using methods described by Viklund et al. (2022). Papayanol was synthesized in our laboratory from racemic grandisol according to the method described by Zarbin et al. (2010). Grandisyl acetate and fragranyl acetate in a ratio of 70:30 were also synthesized at our laboratory (see details in the Supplementary Information).

### Field Trapping Studies to Investigate Chemical Communication Interactions Between the Species

Field studies were conducted in 2017*–*2018 to investigate pheromone-mediated interactions among the *Polygraphus* species in Sweden. All experiments were conducted in spruce dominated forests at locations around Sundsvall in central Sweden. GPS coordinates of these locations can be found in the Supplementary Information, Table S1. Additional field experiments were conducted in 2019–2021 to further investigate the pheromone of *P. subopacus* and *P. poligraphus*. These experiments are described in the Supplementary Information.

“Wick-baits” were used as dispensers. 12.5 mg of a test compound was dissolved in 4 mL of *n*-nonane in a 4 mL glass vial, from which it was allowed to evaporate through a teflon tube (6 cm × 1.5 mm i.d.) lined with cotton yarn and inserted through a drilled hole in the lid of the vial (Birgersson et al. 2012; Viklund et al. 2021). The expected release rate, based on previous studies with compounds of similar molecular weights in a fume hood at the laboratory (22–25 °C, air flow 0.5–0.6 m/s) was 0.46 mg/day (Viklund et al. 2019).

Traps were set up along lines (replicates) with 30 m between the traps and 50 m between the lines. All traps were positioned at least 20 m into the forest. Black Ecotraps (Fytofarm Ltd., Bratislava, Slovak Republic) were used with collection jars for dry catches of living insects, and dispensers were hung just above the collection jars. The dispensers were in this way positioned around 80–90 cm above the ground. Treatments were randomly assigned to their positions within each replicate line in a randomized complete block design. In the 2017 experiment, traps were emptied once per week and treatments were not rotated among positions within each line. In the 2018 experiment, traps were checked and emptied twice per week and treatments rotated among trap positions within lines each time. Captured beetles were stored in a freezer (− 18 °C) until the insects could be counted and identified. Due to the large numbers of beetles collected in 2017, all *Polygraphus* beetles were counted but only a subsample of 50 individuals per trap per week was processed to determine species and sex. In the 2018 field experiment all specimens of *Polygraphus* were counted and identified to species and sex. Species identification and sex determination were done using a stereomicroscope with 14–90 × magnification, according to the methods described by Lekander (1959).

In 2017, we tested five treatments: (1) (*Z*)-DMCHE, (2) *rac*-grandisol, (3) (–)-(*R*)-terpinen-4-ol (99% ee), (4) (*Z*)-DMCHE combined with (–)-(*R*)-terpinen-4-ol (99% ee) and (5) (*Z*)-DMCHE combined with *rac*-grandisol. Treatments were replicated 10X in randomized complete blocks with a different location for each block. The field experiment was conducted from 29 June until 8 August 2017 but due to the large number of beetles collected in some locations we stopped collecting beetles after: 1 week at one location, 2 weeks at four locations, 4 weeks at one location and 5 weeks at another location. Three locations were excluded from the analysis due to missing data. Thus, data from seven blocks were used in the analysis.

In 2018, we tested four treatments: (1) *rac*-grandisol, (2) (–)-(*R*)-terpinen-4-ol (99% ee), (3) *rac*-grandisol in combination with (–)-(*R*)-terpinen-4-ol (99% ee) and (4) control (*n*-nonane) to confirm previous results that indicated the combination of both compounds increased catches of *P. punctifrons* and *P. poligraphus* compared to either compound by itself (Rahmani et al. 2019). Treatments were replicated in three lines at one site. The traps were emptied twice per week and treatments rotated among positions each time. The field experiment was conducted from 2 August until 7 September 2018.

### Statistical Analysis

All statistical analysis was conducted in R Studio (v.2024.09.0 + 375). The number of beetles caught in traps with different treatments were analysed using generalized linear mixed-effects models (GLMMs, package glmmTMB, Brooks et al. 2017). Treatments were designated as fixed effect while blocks (in 2017) and date of emtpying (in 2017 and 2018) were designated as random effects to account for pseudo-replicated sampling design. Blocks were not included in the 2018 models since all replicates were at the same location. Separate models were created for *P. poligraphus*, *P. punctifrons* and *P. subopacus*. The baseline treatment was set to: (–)-(*R*)-terpinen-4-ol (99% ee) for *P. poligraphus, rac*-grandisol for *P. punctifrons* and (Z)-DMCHE for *P. subopacus*. These compounds are known attractants for each of the three species, and the models could thus be used to compare if other treatments were more or less attractive.

The models were fit using negative binomial error distribution to improve the model fit and compliance with residual diagnostics (compared to the Poisson distribution). The model assumptions were tested with quantile residual plots, dispersion and zero-inflation tests (package DHARMa, Hartig 2024). The models were further compared using Akaike Information Criterion (AIC). The model for *P. subopacus* (but not the other species) showed evidence of zero-inflation (*P* = 0.008). Fitting a zero-inflated model for *P. subopacus* solved the problem and resulted in a slightly better fit (lower AIC value).

Estimated marginal means (the predicted mean for each treatment group adjusted for random effects) were derived from the model coefficients (emmeans package, Lenth 2024) and are presented in Tables 2–3 with 95% confidence intervals. In some cases, the models could not properly estimate parameters for specific treatment groups due to a lack of variability within these groups (i.e. non-attractive treatments with all-zero trap catches). In these cases, results were interpreted qualitatively and no confidence intervals are given for these treatment groups in the tables.

## Results

### Newly Identified Compounds Emitted by *P. punctifrons*

**Fig. 1:**
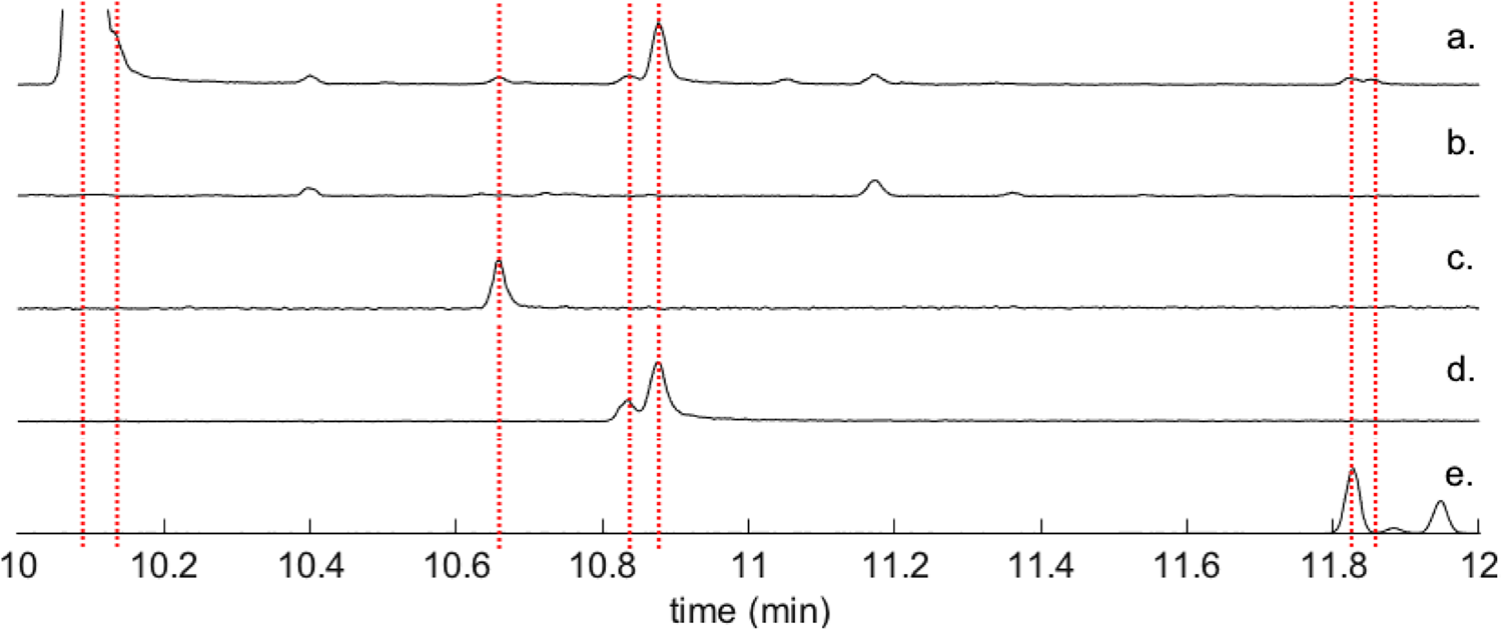
Chromatograms showing a) a representative *P. punctifrons* male, b) a representative *P. punctifrons* female, c) a synthetic reference of geraniol, d) a synthetic reference containing two diastereomers of papayanol and e) a synthetic reference of grandisyl acetate and fragranyl acetate, at a ratio of 70:30. Insects were sampled for 1 h with a pink SPME fiber. The dotted lines represent (from left): grandisol, fragranol, geraniol, two isomers of papayanol, grandisyl acetate and a compound which was not identified

The SPME–GC–MS studies of *P. punctifrons* resulted in the identification of some new compounds which had previously not been found to be emitted by this species (Table 1). Unless otherwise indicated, identifications were made by matches of mass spectra and retention times with commercially-obtained or laboratory-synthesized standards. Three females and five males of *P. punctifrons* started boring into the bark. Several male-specific compounds were identified in addition to the two compounds which have previously been reported, (+)-(*1R,2S*)-grandisol and (*–*)-(*R*)-terpinen-4-ol (Rahmani et al. 2019). The novel compounds were fragranol, geraniol, two isomers of papayanol, grandisyl acetate and a compound which was not identified (Fig. 1). Benzyl alcohol and bezaldehyde were also found to be emitted by *P. punctifrons* males. The stereochemistry of the papayanol isomers and grandisyl acetate were not determined. All these compounds were seen emitted from at least three males and not from any of the females or from the background samples collected from manually drilled holes in the spruce bark. Terpinen-4-ol was present in SPME samples from males as previously shown (Rahmani et al. 2019) although in this study, it was also seen in small amounts in samples from the background and consequently also from the females. The amount of terpinen-4-ol was many times larger in samples from the males based on GC peak areas. Another compound which generated a larger GC peak in SPME samples from males compared to females was α-terpineol, although the difference was not as large as for terpinen-4-ol.

For *P. poligraphus*, only two males and one female started boring into the bark. 2-phenyl ethanol, benzyl alcohol and benzaldehyde were observed from the males in addition to compounds which have previously been identified from *P. poligraphus*. However we could not really confirm whether these compounds were sex-specific since the replicates were too few.

### Unidentified Compounds in *P. punctifrons* and *P. subopacus*

**Fig. 2:**
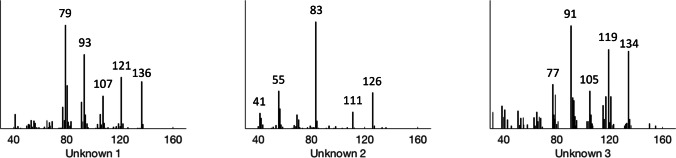
Unidentified compounds in *P. subopacus* (Unknown 1 and 2 with retention times 6.72 min and 6.74 min) and *P. punctifrons* (Unknown 3 with retention time 11.86 min). The GC program was 50(2)—10→250(5) and the column was an HP5-MS

The male-specific GC peak at retention time 11.86 min (Fig. 1) which was observed in SPME collections from *P. punctifrons,* could not be identified as there were no suggestions from the MS library which seemed plausible. The mass spectrum of this compound can be seen in Fig. 2, together with the two unidentified compounds which were found previously in *P. subopacus* (Viklund et al. 2021).

### SPME–GC–MS/EAD Analyses

**Fig. 3:**
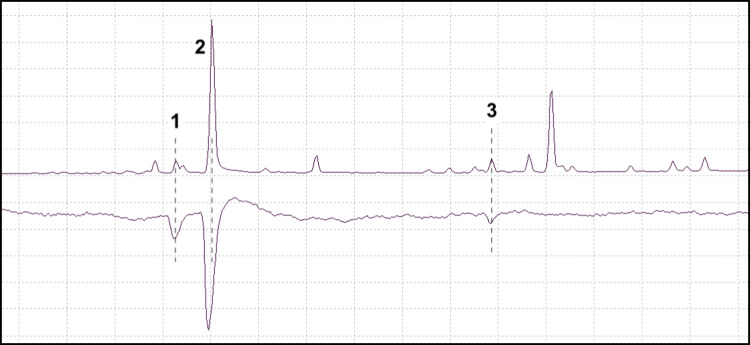
GC-EAD analysis: the SPME sampling of a *P. subopacus* male boring into the bark of a Norway spruce (TIC, upper trace) and the response from a *P. subopacus* female antenna (lower trace). Antennal stimulants were grandisol (1), (*Z*)-DMCHE (2), and a host tree compound (3) longicyclene, according to the NIST14 MS library. Grandisol and (*Z*)-DMCHE are produced by males of *P. subopacus* (Viklund et al. 2021) and longicyclene is a known volatile of Norway spruce (Wajs et al. 2007)

A representative chromatogram and EAD response from *P. subopacus* is shown in Fig. 3. *P. subopacus* responded to the insect-produced grandisol, (*Z*)-DMCHE and a host tree compound with a mass spectrum similar to longicyclene according to the NIST 14 MS library.

**Fig. 4:**
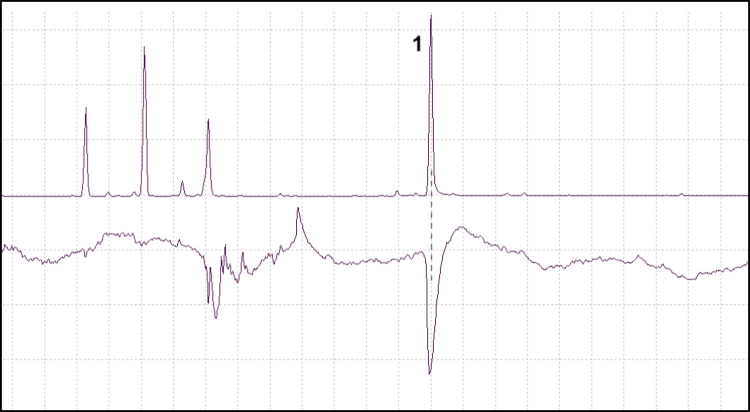
GC-EAD analysis: the SPME sampling from a *P. punctifrons* male boring into the bark of a Norway spruce (TIC, upper trace) and the response from a *P. puncifrons* male antenna (lower trace). The antenna responded to the male-specific compound (+)-(*1R,2S*)-grandisol (1)

Antennae of *P. punctifrons* exposed to SPME collections were also investigated in SPME–GC–MS/EAD analyses, although this species was difficult to analyze since the EAD baseline was somewhat noisy and unstable. However, in some individuals, antennal responses to the insect-specific (+)-(*1R,2S*)-grandisol, the main pheromone compound, could be seen (Fig. 4).

**Fig. 5:**
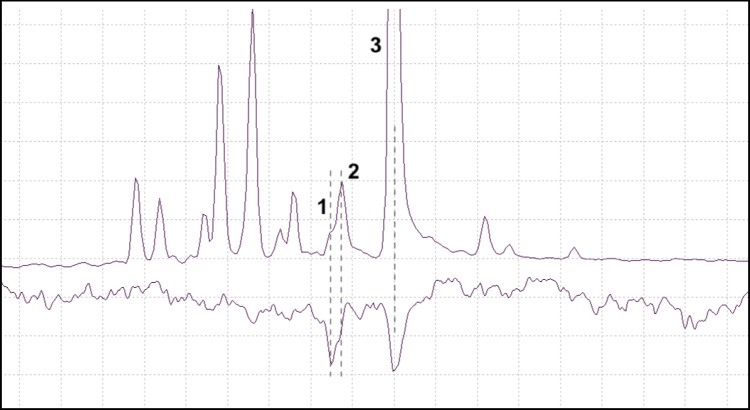
GC-EAD analysis: the SPME sampling of a *P. subopacus* male boring into the bark of a Norway spruce (TIC, upper trace) and the response from a *P. poligraphus* male antenna (lower trace). The antenna responded to the male-specific compounds grandisol (1), (*Z*)-DMCHE (3) and in some cases, fragranol (2)

**Fig. 6:**
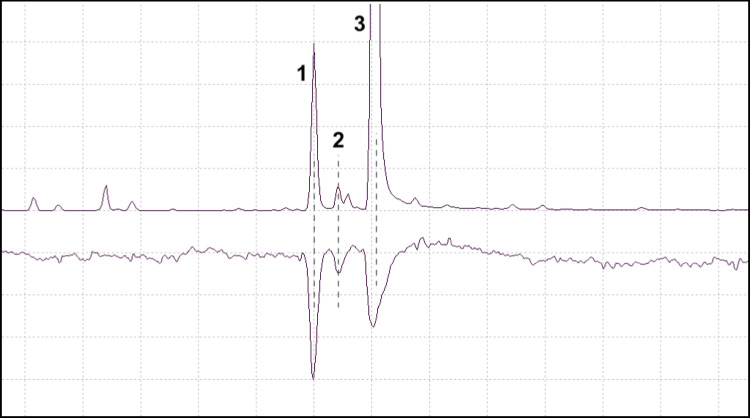
GC-EAD analysis: the SPME sampling of a *P. punctifrons* male boring into the bark of a Norway spruce (TIC, upper trace) and the response from a *P. poligraphus* female antenna (lower trace). The antenna responded to (–)-(*R*)-terpinen-4-ol (1), α-terpineol (2) and (+)-(1*R*,2*S*)-grandisol (3)

When *P. poligraphus* antennae were tested with SPME collections from *P. subopacus* males boring into a Norway spruce stem, responses could be seen for the male-specific compounds grandisol, (*Z*)-DMCHE, and in some cases, fragranol (Fig. 5). The antennal response to grandisol was nearly as strong as the response to (*Z*)-DMCHE, although the latter appeared to be present in much larger quantities in the SPME sample, based on GC peak areas (TIC). *P. poligraphus* antennae were also tested with SPME collections from *P. punctifrons* males, where they responded to the male-specific compounds (*–*)-(*R*)-terpinen-4-ol, (+)-(*1R,2S*)-grandisol and the host tree compound α-terpineol (Fig. 6).

### GC–MS/EAD Analyses Using a Chiral Column

**Fig. 7:**
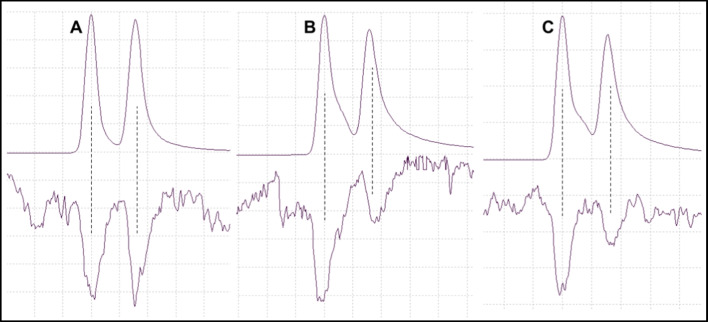
GC-EAD analyses of racemic grandisol (TIC, upper trace) with responses from female *Polygraphus* spp. antenna (lower trace). A: *P. poligraphus*, B: *P. punctifrons*, C: *P. subopacus*

Good quality responses were obtained from one male and four females of *P. poligraphus*, two males and four females of *P. punctifrons* and two females of *P. subopacus*. Representative chromatograms and GC-EAD responses are shown in Fig. 7. All three species responded to both enantiomers of grandisol. In *P. poligraphus*, the response was equally strong for both enantiomers, whereas in *P. punctifrons* and *P. subopacus*, the antenna response was strongest for the first eluting enantiomer. According to previous studies, the enantiomer which elutes first on a Beta DEX™ 225 column is the (–)-(*1S,2R*)-enantiomer (Rahmani et al. 2019).

### Sex-Specific Compounds Emitted from Swedish *Polygraphus* Species and Russian *P. proximus*

Compounds which have been identified in *P. poligraphus*, *P. punctifrons*, *P. subopacus* and *P. proximus* are summarized in Table 1, along with the EAG response for each species (if available).

**Table 1:** Compounds identified from volatiles emitted by *P. poligraphus*, *P. punctifrons*, *P. subopacus* and *P. proximus* colonising stem sections, together with EAG responses to these compounds if available. M = present in males. F = present in females/(F) = possibly present in females. EAG responses: 0 = none, X = weak, XX = strong,—Not determined. 0/X, X/XX = response varies. *Denotes data which has been reported previously (Viklund et al. 2019, 2021, 2022)

	Sex-specific compounds				EAG response		
	*P. poligraphus*	*P. punctifrons*	*P. subopacus*	*P. proximus*	*P. poligraphus*	*P. punctifrons*	*P. subopacus*
3-Methyl-3-buten-1-ol			M*	M*	0*	-	0*
3-Methyl-2-buten-1-ol			M*	M*	0	-	0*
3-Methyl-2-butenal			M*	M*	0	0	0*
1-Hexanol				F*	0*	-	X*
Unknown 1			M*		-	-	-
Unknown 2			M*		-	-	-
Benzaldehyde	M^f^(F)	M	M(F)*	MF*	-	-	0*
Benzyl alcohol	M^f^	M	M*	M*	-	-	0/X*
2-Phenyl ethanol	M^f^				X	-	-
(–)-(*R*)-Terpinen-4-ol	M*	M*			XX*	X	X
( +)-(*S*)-Terpinen-4-ol	M*				XX*	X	X
*cis*-Sabinene hydrate	M^a^*				-	-	-
*trans*-Sabinene hydrate	M^a^*				0/X^e^*	-	0^e^
Grandisol		M*	M^a^*		X/XX^c^	XX^c^	XX^c^*
Fragranol		M^a^	M^a^*	M^a^*	0/X^c^	X^c^	0/X^c^*
γ -Isogeraniol			M*	M*	-	-	0*
(*Z*)-DMCHE			M*	M*	X	X/XX	XX*
(*E*)-DMCHE			M*		0	X/XX	X/XX*
Geraniol		M		M*	0	0	0*
(*Z*)-DMCHA			M*	M*	X	-	0/X*
(*E*)-DMCHA			M*	M*	0/X	-	X*
Geranial			M*	M*	0^d^	0^d^	0^d^*
Papayanol		M^ab^		M^ab^*	-	-	-
Grandisyl acetate		M^a^			-	-	-
Unknown 3		M			-	-	-

^a^ The chirality of the insect-specific compound was not determined. ^b^ Two isomers of papayanol were seen in *P. punctifrons*, whereas only one could be seen in *P. proximus*. ^c^ Racemic compounds were used in the EAG analyses. ^d^ Citral was used for the EAG analysis. ^e^ ( +)-*trans*-sabinene hydrate was used in the EAG analysis. ^f^ Based on two male individuals only

### Field Studies to Investigate Semiochemical Interactions Between the Species

In 2017 (Table 2), traps baited with *rac*-grandisol caught 100% *P. punctifrons* and traps baited with (–)-(*R*)-terpinen-4-ol caught 100% *P. poligraphus,* which reflects previous studies (Rahmani et al. 2019; Viklund et al. 2019). However, traps baited with (*Z*)-DMCHE were not species-specific as the catches consisted of both *P. subopacus* (91%) and *P. poligraphus* (9%). This also reflects previous results (Viklund et al. 2021). For all three species, the baseline treatment significantly influenced beetle catches (*P* < 0.001). For *P. punctifrons,* the addition of (*Z*)-DMCHE to *rac*-grandisol resulted in significantly lower trap catches when compared to *rac*-grandisol alone (*P* < 0.001). In fact, (*Z*)-DMCHE reduced trap catches of *P. punctifrons* to near-zero and thus acted as a repellant for *P. punctifrons* (Table 2). For *P. poligraphus*, the addition of (*Z*)-DMCHE to (–)-(*R*)-terpinen-4-ol increased trap catches significantly (*P* < 0.001). For *P. subopacus*, the addition of *rac*-grandisol to (*Z*)-DMCHE reduced trap catches significantly (*P* = 0.007) while the addition of (–)-(*R*)-terpinen-4-ol did not significantly affect the trap catches.

**Table 2:** Results from the field study in 2017 that investigated semiochemical interactions among the *Polygraphus* species in Sweden

Treatment	Total number of *Polygraphus*	Species in subsample (50 individuals)	Estimated number of insects Mean per trap per week, (95% CI)		
			*P. punctifrons*	*P. poligraphus*	*P. subopacus*
Racemic grandisol	9622	100% *P. punctifrons*	373 (156, 890)	0	0
(–)-Terpinen-4-ol (99% ee)	3722	100% *P. poligraphus*	0 (0, 0)	197 (114, 338)	0
(*Z*)-DMCHE	1786	91% *P. subopacus*, 9% *P. poligraphus*	0 (0, 0)	8 (5, 14)	67 (39, 114)
Racemic grandisol + (*Z*)-DMCHE	1032	79% *P. subopacus,* 16% *P. poligraphus*, 6% *P. punctifrons*	2 (1, 5)	8 (4, 14)	33 (19, 57)
(–)-Terpinen-4-ol (99% ee) + (*Z*)-DMCHE	14,263	94% *P. poligraphus*, 6% *P. subopacus*	0	744 (444, 1246)	39 (23, 68)

This study was conducted from 29 June until 8 August 2017. There were seven blocks in the experiment. For each treatment, the estimated marginal mean per trap per week (adjusted for random effects) is shown with a 95% confidence interval (95% CI)

In 2018 (Table 3), the results from previous studies (Rahmani et al. 2019) could be confirmed, since the combination *rac*-grandisol and (–)-terpinen-4-ol (99% ee) increased the trap catches of *P. punctifrons* compared to *rac*-grandisol alone (*P* < 0,001). The combination was also more attractive to *P. poligraphus* than (–)-terpinen-4-ol (99% ee) alone (*P* < 0.001).

**Table 3:** Results from the field study in 2018, aimed at investigating interactions between pheromone components of *P. punctifrons* and *P. poligraphus*

Treatment	Total number of beetles			Mean per trap per rotation, (95% CI)		
	*P. punctifrons*	*P. poligraphus*	*P. subopacus*	*P. punctifrons*	*P. poligraphus*	*P. subopacus*
Racemic grandisol	1455	2	0	19 (7, 56)	0 (0, 0)	0
(–)-Terpinen-4-ol (99% ee)	0	950	0	0	20 (8, 46)	0
Racemic grandisol + (–)-terpinen-4-ol (99% ee)	2649	3148	0	41 (14, 120)	73 (32, 169)	0
Control (*n*-nonane)	0	3	0	0	0 (0, 0)	0

This study was conducted from 2 August until 7 September 2018. Traps were emptied and rotated twice per week. There were three replicates of each treatment and all traps were at the same location. For each treatment, the estimated marginal mean per trap per rotation (adjusted for random effects) is shown with a 95% confidence interval (95% CI)

## Discussion

Our novel SPME–GC–MS studies of *P. punctifrons* revealed male-specific compounds which have previously not been reported. These were benzaldehyde, benzyl alcohol, fragranol, geraniol, grandisyl acetate, two isomers of papayanol and an unidentified compound. SPME samples from *P. punctifrons* males also reveled that α-terpineol generated a larger GC peak compared to samples from the background or the females. Novel SPME–GC–MS studies of *P. poligraphus* revealed 2-phenyl ethanol and benzyl alcohol in the emissions from two males.

To evaluate the bioactivity of male-specific compounds emitted by *P. subopacus* and *P. punctifrons*, SPME–GC–MS/EAD was used and revealed antenna responses to grandisol, fragranol, (*Z*)-DMCHE, and (*R*)-(–)-terpinen-4-ol. However, (*E*)-DMCHE and (*Z*)-DMCHE coeluted for the GC-column and temperature program used. Two host tree compounds, α-terpineol and longicyclene based on their mass spectra, also produced responses from the antennae of *P. poligraphus* and *P. subopacus* respectively. As the response to grandisol was particularly strong in all three *Polygraphus* species, GC–MS/EAD with a chiral column was performed and we could show that all three species were able to detect both enantiomers of grandisol.

EAG studies of *P. poligraphus* and *P. subopacus* were used to further assess the bioactivity of male-specific compounds and the results gave that the antenna of these species could not detect geraniol, geranial, 3-methyl-3-buten-1-ol, 3-methyl-2-buten-1-ol and 3-methyl-2-butenal, which are male-specific compounds emitted by *P. subopacus* and/or *P. proximus*. *P. punctifrons* antennae could not detect geraniol, geranial and 3-methyl-2-butenal however, this species was also the most difficult to conduct EAG and EAD studies on. These compounds could thus be excluded from further studies, as they were considered not to be bioactive in the *Polygraphus* species which are present in Sweden. Benzyl alcohol and benzaldehyde were seen in emissions from all four *Polygraphus* species investigated in this work and previous work (Viklund et al. 2022, 2021)*,* and were thus considered to not play a role in the species-specificity of their aggregation pheromones. Benzaldehyde is known as a host tree volatile in Norway spruce (Wajs et al. 2007) and benzyl alcohol may be emitted by bark-beetle related fungi (Kandasamy et al. 2016). *Polygraphus* spp. have been associated with several species of fungi (Pashenova et al. 2018; Jankowiak et al 2014; Rollins et al. 2001; Krokene & Solheim 1996; Baranchikov et al. 2017). It has been suggested that geraniol and also γ-isogeraniol (which was seen in emissions from *P. proximus* and to some extent also from *P. subopacus* males) are biosynthetic precursors of (*Z*)-DMCHE, (*E*)-DMCHE and grandisol (Byers et al. 2013; Thompson and Mitlin 1979).

All three Swedish species could, however, detect grandisol, fragranol, the enantiomers of terpinen-4-ol and one or both stereoisomers of DMCHE. Both DMCHA stereoisomers could be detected by the two species which they were tested on, ie *P. poligraphus* and *P. subopacus*. Additionally, *P. subopacus* could detect 1-hexanol and *P. poligraphus* could detect 2-phenyl ethanol. Thus, all these compounds were candidates for use in field studies. Stereoisomers of DMCHE and DMCHA as well as grandisol are known to be pheromone components in many other species of weevils (Ambrogi et al. 2012; Booth et al. 1983; Byers et al. 2013; Hedin et al. 1997; Hibbard and Webster 1993; Innocenzi et al. 2001; Marques et al. 2011; Rodriguez-Saona et al. 2020; Szendrei et al. 2011).

A major finding of our field trapping studies was that (*Z*)-DMCHE repels *P. punctifrons* from traps baited with racemic grandisol. This may explain why *P. punctifrons* is not attracted by the aggregation pheromone of *P. subopacus*, although males of both species emit grandisol. For *P. subopacus*, no species-specific lure composition could be found, although several male-specific compounds and combinations of compounds were tested in field studies (Supplementary Information, Tables S2–S4). All the combinations which were attractive to *P. subopacus* were also attractive to *P. poligraphus*. There still might be an unidentified compound in *P. subopacus*’ aggregation pheromone which would repel* P. poligraphus*, making the aggregation pheromone of *P. subopacus* species-specific, but this needs to be investigated further. The low chemical stability of (*Z*)- and (*E*)-DMCHA (Henson et al. 1976) caused these aldehydes to degrade quickly in our field study in 2020, making the results somewhat questionable. However, in 2021, the two aldehydes still did not affect the by-catch of *P. poligraphus* in the field despite addition of BHT as a stabilizer. For *P. poligraphus*, the only compounds which increased the attraction of the aggregation pheromone (–)-(*R*)-terpinen-4-ol were unexpectedly found to be (*Z*)-DMCHE and grandisol – the main pheromone components of *P. subopacus* and *P. punctifrons* respectively. 1-Hexanol, which is emitted by *P. proximus* females, also increased trap catches of *P. poligraphus* in our 2019 field study. However, considering the overall small trap catches in 2019 together with the fact that *P. poligraphus* could not detect 1-hexanol in EAG studies, these results should be interpreted with caution.

 When analysing males of *P. punctifrons,* we registered (–)-(*R*)-terpinen-4-ol as well as (+)-(*1R,2S*)-grandisol, but these two compounds in combination are also attractive to *P. poligraphus.* (–)-(*R*)-terpinen-4-ol is a host tree compound which we could see in small amounts in SPME samples from the spruce tree background and from boring *P. punctifrons* females. However we identified much higher amounts from boring *P. punctifrons* males based on GC peak area and this leads to that (–)-(*R*)-terpinen-4-ol is indeed produced by the males. As the combination of *rac*-grandisol and (–)-(*R*)-terpinen-4-ol is attractive to both *P. punctifrons* and *P. poligraphus*, we hypothesize that an additional compound is required to make the aggregation pheromone of *P. punctifrons* species-specific. In light of our new SPME–GC–MS/EAD studies, the results indicate that this compound may be α-terpineol. α-Terpineol is, like (–)-(*R*)-terpinen-4-ol, present in small amounts in the spruce background (Wajs et al. 2007) but in larger amounts in our SPME samples from males of *P. punctifrons*. We know from previous studies that α-terpineol is a repellant for *P. poligraphus* (Viklund et al. 2019). Different *Polygraphus* species are occasionally found on the same host and whether cross-attraction between species is present in the *Polygraphus* genus may be investigated in future studies.

*P. proximus* was not directly included in this work; however, we hypothesize that there should be a compound emitted by *P. proximus* which would repel *P. subopacus* as (*Z*)-DMCHE is the main component of the aggregation pheromone of both species. 1-Hexanol which is emitted by females of *P. proximus* did not repel *P. subopacus* in the field (Supplementary Information, Table S2). It is possible that papayanol is a repellent for both *P. poligraphus* and *P. subopacus*, which may prevent the attraction of these species to the aggregation pheromone of *P. proximus* and perhaps *P. punctifrons*. Unfortunately, we were not able to test papayanol on EAG or in field studies. It is also possible that the stereochemistry of papayanol, fragranol and grandisol differ between the *Polygraphus* species. Investigating the stereochemistry of these compounds would thus be an interesting objective for future studies. Efforts should also be made to identify the three unidentified compounds in *P. subopacus* and *P. punctifrons.* However, the lack of antennal response during SPME–GC–MS/EAD studies at the GC-retention time of these compounds may indicate that they are not biologically active. Although *P. poligraphus* and *P. subopacus* could not detect 3-methyl-2-buten-1-ol in EAG studies, *P. proximus* likely can sense it as they were attracted to it in previous field studies (Viklund et al. 2022). In the related species *Polygraphus rufipennis*, a similar compound, 3-methyl-3-buten-1-ol has been identified as an aggregation pheromone (Bowers et al. 1991).

In conclusion, species-specific pheromone baits exist for *P. poligraphus* and *P. punctifrons,* as (–)-(*R*)-terpinen-4-ol catches *P. poligraphus* and racemic grandisol catches *P. punctifrons*. Since there are other male-specific compounds present in the volatiles emitted from these two species and as aggregation pheromones rarely consist of only one compound, it is likely that these pheromone baits can be made more effective. For *P. subopacus*, no species-specific pheromone blend has yet been developed, despite the several field studies in this paper and its Supplementary Information, as well as in previous work (Viklund et al. 2021, 2022). The large number of male-specific compounds which have been identifed in this species contribute to the complexity of this problem. In Siberia, a blend of (*Z*)-DMCHE and (*E*)-DMCHE in a ratio of 10:1 can be used to catch *P. subopacus* specifically (Viklund et al. 2022) but if this blend is used in traps in Sweden, *P. poligraphus* is also caught in the traps. For *P. proximus*, the composition of its aggregation pheromone has not been completely determined. (*Z*)-DMCHE appears to be the major pheromone component, but baiting traps with this compound alone results in large by-catches of *P. subopacus.* 3-Methyl-2-buten-1-ol appears to attract *P. proximus* specifically, but if it is combined with (*Z*)-DMCHE, *P. subopacus* is also caught in the traps (Viklund et al. 2022). In the light of our new SPME–GC–MS, EAG and SPME–GC–MS/EAD studies, we believe that the key to species-specificity of the aggregation pheromones in *P. subopacus* and *P. proximus* may involve papayanol, fragranol or grandisol. In that case, the stereochemistry of these compounds needs to be considered as different enantiomers may be used by different species.

## Supplementary Information

Below is the link to the electronic supplementary material.

Supplementary file1 (DOCX 47 KB)

## Data Availability

Data is provided within the manuscript or supplementary information files. Should any raw data files be needed in another format, they are available from the corresponding author upon reasonable request.

## References

[CR1] Ambrogi BG, Palacio-Cortés AM, Zarbin PHG (2012) Identification of male-produced aggregation pheromone of the curculionid beetle *Sternechus**subsignatus*. J Chem Ecol 38:272–277. 10.1007/s10886-012-0080-3 22383053 10.1007/s10886-012-0080-3

[CR2] Bandeira PT, Fávaro CF, Francke W, Bergmann J, Henrique P, Zarbin G (2021) Aggregation pheromones of weevils (Coleoptera: Curculionidae): Advances in the identification and potential uses in semiochemical-based pest management strategies. J Chem Ecol 47:968–986. 10.1007/s10886-021-01319-1 34671912 10.1007/s10886-021-01319-1

[CR3] Baranchikov YN, Demidko DA, Pashenova NV, Pertsovaya AA, Petko VM (2017) Ophiostomal fungi in the trunk of Siberian fir increase its attractiveness for bark beetles. Current Mycology in Russia. Moscow: National Mycological Academy. 6:355–357 (in Russian)

[CR4] Birgersson G, Dalusky MJ, Espelie KE, Berisford W (2012) Pheromone production, attraction, and interspecific inhibition among four species of *Ips* bark beetles in the southeastern USA. Psyche: J Entomol 2012(6):1–14. 10.1155/2012/532652

[CR5] Blomquist GJ, Figueroa-Teran R, Aw M, Song M, Gorzalski A, Abbott NL, Chang E, Tittiger C (2010) Pheromone production in bark beetles. Insect Biochem Mol Biol 40:699–712. 10.1016/j.ibmb.2010.07.013 20727970 10.1016/j.ibmb.2010.07.013

[CR6] Booth DC, Phillips TW, Claesson A, Silverstein RM, Lanier GN, West JR (1983) Aggregation pheromone components of two species of *Pissodes* weevils (Coleoptera: Curculionidae): Isolation, identification and field activity. J Chem Ecol 9:1–12. 10.1007/BF00987766 24408615 10.1007/BF00987766

[CR7] Bowers WW, Gries G, Borden JH, Pierce HD Jr (1991) 3-methyl-3-buten-1-ol: An aggregation pheromone of the four-eyed spruce bark beetle, *Polygraphus**rufipennis* (Kirby) (Coleoptera: Scolytidae). J Chem Ecol 17:1989–2002. 10.1007/BF00992583 24258493 10.1007/BF00992583

[CR8] Bowers WW, Borden JH, Raske A (1996) Incidence and impact of *Polygraphus**rufipennis* (Coleoptera: Scolytidae) in Newfoundland. For Ecol Manag 89:173–187. 10.1016/S0378-1127(96)03850-9

[CR9] Brooks ME, Kristensen K, van Benthem KJ, Magnusson A, Berg CW, Nielsen A, Skaug HJ, Maechler M, Bolker BM (2017) glmmTMB balances speed and flexibility among packages for zero-inflated generalized linear mixed modeling. R J 9(2):378–400. 10.32614/RJ-2017-066

[CR10] Byers JA, Birgersson G, Francke W (2013) Aggregation pheromones of bark beetles, *Pityogenes**quadridens* and *P.**bidentatus*, colonizing Scotch pine: olfactory avoidance of interspecific mating and competition. Chemoecology 23:251–261. 10.1007/s00049-013-0139-9

[CR11] De la Peña E, Kinkar M, Vos S. (2020) Pest survey card on *Polygraphus**proximus*. EFSA supporting publications. European Food Safety Authority, Parma. 10.2903/sp.efsa.2020.EN-1780 Accessed 9 April 2024

[CR12] Ehnström B, Axelsson R (2002) Insektsgnag i bark och ved. ArtDatabanken SLU (in Swedish)

[CR13] Ephrussi B, Beadle GW (1936) A technique of transplantation for Drosophila. Am Nat 70(728):218–225. 10.1086/280658

[CR14] EPPO (2014) Pest risk analysis for *Polygraphus**proximus*. EPPO, Paris. http://www.eppo.int/QUARANTINE/Pest_Risk_Analysis/PRA_intro.htm Accessed 30 March 2024.

[CR15] Fettig CJ, Hilszczański J (2015) Management strategies for bark beetles in coniferous forests. In: Vega FE, Hofstetter RW (eds) Bark Beetles. San Diego: Academic Press, pp 585–513. 10.1016/B978-0-12-417156-5.00014-9

[CR16] Grégoire J-C, Raffa, KF, Lindgren BS (2015) Economics and politics of bark beetles. In: Vega FE, Hofstetter RW (eds) Bark Beetles. San Diego: Academic Press, pp 585–513. 10.1016/B978-0-12-417156-5.00015-0

[CR17] Hartig F (2024) DHARMa: Residual diagnostics for hierarchical (multi-level/mixed) regression models. R package version 0.4.7, https://CRAN.R-project.org/package=DHARMa. Accessed 13 Dec 2024

[CR18] Hedin PA, Dollar DA, Collins JK, Dubois JG, Mulder PG, Hedger GH, Smith MW, Eikenbary RD (1997) Identification of male pecan weevil pheromone. J Chem Ecol 23(4):965–977. 10.1023/B:JOEC.0000006382.70034.66

[CR19] Henson RD, Bull DL, Ridgway RL, Ivie GW (1976) Identification of the oxidative decomposition products of the boll weevil pheromone, Grandlure, and the determination of the fate of Grandlure in soil and water. J Agric Food Chem 24(2):228–231. 10.1021/jf60204a019 943430 10.1021/jf60204a019

[CR20] Hibbard BE, Webster FX (1993) Enantiomeric composition of grandisol and grandisal produced by Pissodes strobi and P. nemorensis and their electroantennogram response to pure enantiomers. J Chem Ecol 19(10):2129–2141. 10.1007/BF00979652 24248564 10.1007/BF00979652

[CR21] Hlásny T, König L, Krokene P, Lindner M, Montagné-Huck C, Müller J, Qin H, Raffa KF, Schelhaas M-J, Svoboda M, Viiri H, Seidl R (2021) Bark beetle outbreaks in Europe: State of knowledge and ways forward for management. Curr Forestry Rep 7:138–165. 10.1007/s40725-021-00142-x

[CR22] Innocenzi PJ, Hall DR, Cross JV (2001) Components of male aggregation pheromone of strawberry blossom weevil, *Anthonomus**rubi* Herbst. (Coleoptera: Curculionidae). J Chem Ecol 27:1203–1218. 10.1023/A:1010320130073 11504023 10.1023/a:1010320130073

[CR23] Jankowiak R, Kolarik M, Bilanski P (2014) Association of *Geosmithia* fungi (Ascomycota: Hypocreales) with pine- and spruce-infesting bark beetles in Poland. Fungal Ecol 11:71–79. 10.1016/j.funeco.2014.04.002

[CR24] Kandasamy D, Gershenzon J, Hammerbacher A (2016) Volatile organic compounds emitted by fungal associates of conifer bark beetles and their potential in bark beetle control. J Chem Ecol 42:952–969. 10.1007/s10886-016-0768-x 27687998 10.1007/s10886-016-0768-x PMC5101256

[CR25] Kerchev IA (2014) On monogyny of the four-eyed fir bark beetle Polygraphus proximus Blandf. (Coleoptera, Curculionidae: Scolytinae) and its reproductive behavior. Entomol Rev 94(8):1059–1066. 10.1134/S0013873814080028

[CR26] Kerchev IA (2020) Interspecific differences of stridulatory signals in three species of bark beetles from the genus Polygraphus Er. (Coleoptera: Curculionidae, Scolytinae) inhabiting the island of Sakhalin. PeerJ 8:8281. 10.7717/peerj.8281 10.7717/peerj.8281 PMC6942674 31915580

[CR27] Krivets SA, Kerchev IA, Bisirova EM, Debkov NM, Chernova NA, Pats EN (2019) 3.2.1 Four-eyed fir bark beetle *Polygraphus**proximus* Blandford, 1984 (Coleoptera, Curculionidae: Scolytinae) in Western Siberia: review of ten years of research of the invasion. In: Gninenko YI (ed) Invasive Dendrophilous Organisms: Challenges and Protection Operations. All-Russian Research Institute of Silviculture and Mechanization of Forestry, Pushkino, pp 87–103

[CR28] Krokene P, Solheim H (1996) Fungal associates of five bark beetle species colonizing Norway spruce. Can J for Res 26:2115–2122. 10.1139/x26-240

[CR29] Lekander B (1959) Der doppeläugige Fichtenbastkäfer *Polygraphus**poligraphus* L. - Ein Beitrag zur Kenntnis seiner Morphologie, Anatomie, Biologie und Bekämpfung. Meddelanden Från Statens Skogsforskningsinstitut 48:1–127 (in German)

[CR30] Lenth R (2024) emmeans: Estimated marginal means, aka least-squares means. R package version 1.10.5, https://CRAN.R-project.org/package=emmeans. Accessed 13 Dec 2024

[CR31] Marques FA, Zaleski SRM, Lazzari SMN, Frensch G, Senhorini GA, Maia BHLNS, Tröger A, Francke W, Iede ET, Mori K (2011) Identification of (*1R, 2S*)-grandisal and (*1R, 2S*)-grandisol in *Pissodes**castaneus* male-produced volatiles: Evidence of a sex pheromone. J Braz Chem Soc 22(6):1050–1055. 10.1590/S0103-50532011000600007

[CR32] Pashenova NV, Kononov AV, Ustyantsev KV, Blinov AG, Pertsovaya AA, Baranchikov YN (2018) Ophiostomatoid fungi associated with the four-eyed fir bark beetle on the territory of Russia. Russ J Biol Invasions 9:63–74. 10.1134/S2075111718010137

[CR33] Patacca M, Lindner M, Lucas-Borja ME, Cordonnier T, Fidej G, Gardiner B, Hauf Y, Jasinevicius G, Labonne S, Linkevicius E, Mahnken M, Milanovic S, Nabuurs G-J, Nagel TA, Nikinmaa L, Panyatov M, Bercak R, Seidl R, Ostrogovic Sever MZ, Socha J, Thom D, Vuletic D, Zudin S, Schelhaas M-J (2022) Significant increase in natural disturbance impacts on European forests since 1950. Glob Change Biol 29:1359–1376. 10.1111/gcb.16531 10.1111/gcb.16531 PMC10107665 36504289

[CR34] Raffa KF, Grégoire J-C, Lindgren BS (2015) Natural history and ecology of bark beetles. In: Vega FE, Hofstetter RW (eds) Bark Beetles. San Diego: Academic Press, pp 1–40. 10.1016/B978-0-12-417156-5.00001-0

[CR35] Rahmani R, Hedenström E, Schroeder M (2015) SPME collection and GC-MS analysis of volatiles emitted during the attack of male *Polygraphus**poligraphus* (Coleoptera, Curcolionidae) on Norway spruce. Z Naturforsch C 70:265–273. 10.1515/znc-2015-5035 26461842 10.1515/znc-2015-5035

[CR36] Rahmani R, Wallin EA, Viklund L, Schroeder M, Hedenström E (2019) Identification and field assay of two aggregation pheromone components emitted by males of the bark beetle *Polygraphus**punctifrons* (Coleoptera: Curculionidae). J Chem Ecol 45:356–365. 10.1007/s10886-019-01056-6 30796678 10.1007/s10886-019-01056-6 PMC6477006

[CR37] Rodriguez-Saona C, Alborn HT, Oehlschlager C, Calco C, Kyryczenko-Roth V, Tewari S, Sylvia MM, Averill AL (2020) Fine-tuning the composition of the cranberry weevil (Coleoptera: Curculionidae) aggregation pheromone. J Appl Entomol 144:417–421. 10.1111/jen.12752

[CR38] Rollins F, Jones KG, Krokene P, Solheim H, Blackwell M (2001) Phylogeny of asexual fungi associated with bark and ambrosia beetles. Mycologia 93(5):991–996. 10.2307/3761761

[CR39] Schroeder M (2023) Granbarkborrens förökningsframgång i dödade träd under sommaren 2022 i sydöstra Småland, Värmland och Uppland/Västmanland. Arbetsrapport. Institutionen för Ekologi, SLU, Uppsala (in Swedish)

[CR40] Schroeder M, Kärvemo S (2022) Rekordstort utbrott av granbarkborre – orsaker och vad man kan göra. Kungliga Skogs- Och Lantbruksakademiens Tidskrift Nr 7:2022 (in Swedish)

[CR41] Schurig V, Leyrer U, Kohnle U (1985) Enantiomer composition and absolute configuration of terpinen-4-ol from the bark beetle *Polygraphus**poligraphus*. Naturwissenschaften 72(4):211. 10.1007/BF01195767

[CR42] Silverstein RM, Young JC (1976) Insects generally use multicomponent pheromones. In: Beroza M (ed) Pest Management with Insect Sex Attractants and Other Behaviour-Controlling Chemicals. ACS Symposium Series no 23. American Chemical Society, Washington DC, pp 1–29

[CR43] Szendrei Z, Averill A, Alborn H, Rodriguez-Saona C (2011) Identification and evaluation of attractants for the cranberry weevil, *Anthonomus**musculus* Say. J Chem Ecol 37:387–397. 10.1007/s10886-011-9938-z 21445566 10.1007/s10886-011-9938-z

[CR44] Thompson AC, Mitlin N (1979) Biosynthesis of the sex pheromone of the male boll weevil from monoterpene precursors. Insect Biochem 9:293–294. 10.1016/0020-1790(79)90008-8

[CR45] Tillman JA, Seybold SJ, Jurenka RA, Blomquist GJ (1999) Insect pheromones – an overview of biosynthesis and endocrine regulation. Insect Biochem Mol Biol 29:481–514. 10.1016/s0965-1748(99)00016-8 10406089 10.1016/s0965-1748(99)00016-8

[CR46] Viklund L, Rahmani R, Bång J, Schroeder M, Hedenström E (2019) Optimizing the attractiveness of pheromone baits used for trapping the four-eyed spruce bark beetle *Polygraphus**poligraphus*. J Appl Entomol 143(7):721–730. 10.1111/jen.12641

[CR47] Viklund L, Bång J, Schroeder M, Hedenström E (2021) Identification of male produced compounds in the bark beetle *Polygraphus**subopacus* and establishment of (*Z*)-2-(3,3-dimethylcyclohexylidene)-ethanol as an aggregation pheromone component. Chemoecology 31:367–376. 10.1007/s00049-021-00358-0

[CR48] Viklund L, Baranchikov Y, Schroeder M, Efremenko A, Demidko D, Hedenström E (2022) Identification of sex-specific compounds in the invasive four-eyed fir bark beetle *Polygraphus**proximus*. Chemoecology 32:183–195. 10.1007/s00049-022-00377-5

[CR49] Wajs A, Pranovich A, Reunanen M, Willför S, Holmbom B (2007) Headspace-SPME Analysis of the Sapwood and Heartwood of *Picea**Abies*, *Pinus**Sylvestris* and *Larix**Decidua*. J Essent Oil Res 19(2):125–133. 10.1080/10412905.2007.9699244

[CR50] Wulff S, Hansson P (2013) Nationell riktad skadeinventering (NRS) 2012. Arbetsrapport 386 2013. Institutionen för skoglig resurshushållning, SLU, Umeå (in Swedish)

[CR51] Wulff S, Roberge C (2023) Nationell Riktad Skadeinventering (NRS) - Inventering av barkborreangrepp i Götaland och Svealand. Arbetsrapport. Institutionen för skoglig resurshushållning 2023–12–08 (in Swedish)

[CR52] Zarbin PHG, Moreira MAB, Haftmann J, Tröger A, Franke S, Kopf J, Mori K, Francke W (2010) 1*R*,2*S*,6*R*)-2-Hydroxymethyl-2,6-dimethyl-3- oxabicyclo[4.2.0]octane, a new volatile released by males of the papaya borer *Pseudopiazurus**obesus* (Col.: Curculionidae. Org Lett 12:2447–2449. 10.1021/ol100074q 20465270 10.1021/ol100074q

