# Supplementary material for: New Insights into the Composition of Aggregation Pheromones in Polygraphus poligraphus, Polygraphus punctifrons, Polygraphus subopacus and Polygraphus proximus
Source: J Chem Ecol. 2025 Feb 7;51(1):25. doi: 10.1007/s10886-025-01577-3 (PMC11805885; doi:10.1007/s10886-025-01577-3)
Supplement: Supplementary file 1 — Supplementary file1 (DOCX 47 KB) [file 10886_2025_1577_MOESM1_ESM.docx]

## SUPPLEMENTARY INFORMATION

**NEW INSIGHTS INTO THE COMPOSITION OF AGGREGATION PHEROMONES IN *Polygraphus poligraphus*, *Polygraphus punctifrons*, *Polygraphus subopacus* AND *Polygraphus proximus***

Journal of Chemical Ecology

LINA VIKLUND, JOAKIM BÅNG, MARTIN SCHROEDER and ERIK HEDENSTRÖM^*^

**Affiliation and email address of the corresponding author:
Eco-Chemistry, Department of Natural Science, Design and Sustainable Development, Mid Sweden University, SE-851 70 Sundsvall, Sweden*

*erik.hedenstrom@miun.se*

CHEMICAL SYNTHESIS

*Synthesis of Grandisyl Acetate and Fragranyl Acetate, 70:30.* A mixture of *rac*-grandisol and *rac*-fragranol was synthesized previous at our laboratory (Rahmani et al. 2019). A few mg of the compounds were further purified by SPE (500 mg SiOH, Chromabond) using *n*-pentane as solvent with an increasing concentration of ethyl acetate (0-15% + 100%). After evaporation of the solvents under Ar, six fractions were obtained of which one contained 70% grandisol and 30% fragranol. 0.5 ml of dichloromethane, one drop of acetyl chloride and two drops of pyridine was added to this fraction. The mixture was stirred until the next day, when it had become brown and transparent. The obtained acetates were purified by liquid-liquid extraction using an additional 0.5 ml of dichloromethane and HCl (1M), 1 ml x 2. The combined aquaous phases were extracted with 1 ml of *n*-pentane, and the organic phases were combined and filtered through MgSO_4._ The solvent was evaporated under Ar, compounds were redissolved in 1 ml of *n*-pentane and remaining impurities were removed by another extraction with distilled water, 1 ml x 3. The organic phase was again dried with MgSO_4_ and the solvent was evaporated, yielding a 70:30 mixture of grandisyl acetate and fragranyl acetate. Grandisyl acetate. MS (EI) m/z: 43(44), 68(100), 93(31), 108(21), 136(3). Fragranyl acetate. MS (EI) m/z: 43(45), 68(100), 93(30), 108(19), 136(2).

FIELD STUDIES

*Field Studies to Further Investigate the Pheromone of P. subopacus and P. poligraphus.* Additional field studies were conducted in order to find additional attractants and repellants for *P. subopacus* and *P. poligraphus*, where the main goal was to reduce by-catches of *P. poligraphus* in traps intended to detect *P. subopacus*. Another goal was to increase trap catches of both species. In all cases, the traps were emptied twice per week and the trap positions were rotated each time the traps were emptied. “Wick-baits” were used as dispensers. In each dispenser, the test compound(s) were dissolved in 4 mL of *n*-nonane. In 2019 and 2020, the loads per dispenser were 25 mg for (*Z*)-DMCHE and 2.5 mg for all other compounds. In 2021 the load of each test compound per dispenser was 12.5 mg. The expected release rates, based on previous studies with compounds of similar molecular weights in a fume hood at the laboratory (22–25 °C, air flow 0.5–0.6 m/s) were 0.09, 0.46, and 0.92 mg/day, respectively, for loads of 2.5, 12.5 and 25 mg (Viklund et al. 2019). All *Polygraphus* beetles which were caught were counted and identified to species and sex.

**2019**. Six treatments were tested: (1) (*Z*)-DMCHE, (2) *rac*-frontalin, (3) 1-hexanol, (4) (*Z*)-DMCHE combined with *rac*-frontalin, (5) (*Z*)-DMCHE combined with 1-hexanol and (6) control traps with *n*-nonane*.* Treatments were replicated in three lines at one location and the experiment was conducted from 1–15 July 2019.

**2020**. 10 treatments were tested: (1) (*Z*)-DMCHE, (2) (*Z*)-DMCHA, (3) (*E*)-DMCHA, (4) benzyl alcohol, (5) (*Z*)-DMCHE combined with (*Z*)-DMCHA, (6) (*Z*)-DMCHE combined with (*E*)-DMCHA, (7) (*Z*)-DMCHE combined with benzyl alcohol, (8) (*Z*)-DMCHE combined with *rac*-grandisol, (9) (*Z*)-DMCHE combined with (*E*)-DMCHE, (*Z*)-DMCHA, (*E*)-DMCHA and *rac*-grandisol, and (10) control traps baited with only *n*-nonane. Treatments were replicated in three lines at one location and the experiment was conducted between 30 July and 8 September 2020.

**2021**. Two studies were conducted. In both cases, the treatments were replicated at three sites (blocks) and the experiments were conducted between 22 July and 1 September 2021. The first study focused on optimizing a pheromone bait for *P. subopacus*, this time with higher evaporation rates of the added compounds. The eight treatments were; (1) (*Z*)-DMCHE, (2) (*E*)-DMCHE, (3) (*Z*)-DMCHA (4) (*E*)-DMCHA, (5) (*Z*)-DMCHE combined with (*E*)-DMCHE, (6) (*Z*)-DMCHE combined with (*Z*)-DMCHA, (7) (*Z*)-DMCHE combined with (*E*)-DMCHA and (8) control traps with *n*-nonane. Butylated hydroxytoluene (BHT) was added to (*Z*)- and (*E*)-DMCHA as a stabilizer, at a ratio of 3% of the weight of the compound.

The other study in 2021 focused on finding a synergist of *P. poligraphus* pheromone and included four treatments: (1) (–)-(*R*)-terpinen-4-ol (99% ee), (2) 2-phenyl ethanol, (3) (–)-(*R*)-terpinen-4-ol (99% ee) combined with 2-phenyl ethanol and (4) control traps with *n*-nonane.

*Locations.* The GPS coordinates of all sites used in the field studies in **2017–2021** are presented in Table S1. In 2017, trap catches from seven sites (blocks) were used in the analysis. All sites were located west of Sundsvall, Sweden. Two sites could be found south of Torpshammar, one in Nedansjö, two north of Stöde and another two south of Stöde. In the field studies in 2018, 2019 and 2020, only one site was used for all replicates. It was located north-west of Sundsvall at Knivtjärn, between Kovland and Holm. In 2021, three locations south of Sundsvall were used; two were at Armsjön and one near Galtström.

**Table S1**. GPS coordinates (WGS 84) of the locations used in the field studies.

| 2017 |  | 2018, 2019 and 2020 | | 2021 |  |
| --- | --- | --- | --- | --- | --- |
| ° N | ° E | ° N | ° E | ° N | ° E |
| 62.457313 | 16.193893 | 62.578640 | 16.897460 | 62.153577 | 17.429370 |
| 62.450877 | 16.323165 |  |  | 62.140361 | 17.390163 |
| 62.497520 | 16.686912 |  |  | 62.160737 | 17.449407 |
| 62.466291 | 16.755471 |  |  |  |  |
| 62.380334 | 16.856035 |  |  |  |  |
| 62.327155 | 16.485809 |  |  |  |  |
| 62.287846 | 16.501259 |  |  |  |  |

*Statistical Analysis of the Field Studies in 2019*–*2021.* Generalized linear mixed models were fit using the glmmTMB package in R Studio. The DHARMa package was used for residual diagnostics and the emmeans package was used to calculate the estimated marginal means (predicted means adjusted for random effects) and their 95% confidence intervals, which are presented in Tables S2–S5. For treatment groups with all-zero trap catches, no confidence intervals are given. Each species was modeled individually. The number of caught beetles was modeled as a function of treatment (fixed effect) with date of emptying (2019–2021) and blocks (2021) as random effects. Unless otherwise stated, a negative binomial error distribution was used in the models. The baseline treatment was set to (*Z*)-DMCHE in these studies, except for the second study in 2021 where the baseline treatment was set to (–)-(*R*)-terpinen-4-ol (99% ee).

**2019.** Traps baited with (*Z*)-DMCHE caught both *P. subopacus* and *P. poligraphus* (Table S2). For *P. poligraphus*, the addition of frontalin reduced trap catches (*P* = 0.028) while the addition of 1-hexanol increased trap catches (*P* < 0.001). For *P. subopacus*, the treatment effect was not statistically significant. Overall, very few beetles were caught in this experiment so the results should be interpreted with caution.

**Table S2** Results from the field study in 2019, aimed at investigating the pheromone of *P. subopacus*

| Treatment | Total number of beetles | | | Mean per trap per rotation, (95% CI) | | |
| --- | --- | --- | --- | --- | --- | --- |
|  | *P. punctifrons* | *P. poligraphus* | *P. subopacus* | *P. punctifrons* | *P. poligraphus* | *P. subopacus* |
| (*Z*)-DMCHE | 0 | 202 | 429 | 0 | 9 (2, 34) | 2 (0, 117) |
| Racemic frontalin | 0 | 11 | 0 | 0 | 1 (0, 3) | 0 |
| 1-Hexanol | 0 | 2 | 0 | 0 | 0 (0, 1) | 0 |
| (*Z*)-DMCHE  + racemic frontalin | 0 | 82 | 309 | 0 | 3 (1, 14) | 2 (0, 83) |
| (*Z*)-DMCHE  + 1-hexanol | 0 | 864 | 595 | 0 | 34 (9, 132) | 3 (0, 160) |
| Control (*n*-nonane) | 0 | 2 | 0 | 0 | 0 (0, 1) | 0 |

This study was conducted from 31 June until 15 July 2019. Traps were emptied and rotated twice per week. Estimated marginal means per trap per rotation (adjusted for random effects) are shown for each treatment, with a 95% confidence interval (95% CI).

**2020**. Once again, both *P. subopacus* and *P. poligraphus* were attracted to traps baited with (*Z*)-DMCHE (Table S3) and the treatment effect was statistically significant for both species (*P* = 0.02 and *P* < 0.001). However, adding other compounds to (*Z*)-DMCHE did not significantly affect the trap catches.

**Table S3** Results from the field study in 2020, aimed at investigating the pheromone of
*P. subopacus*

| Treatment | Total number of beetles | | | Mean per trap per rotation, (95% CI) | | |
| --- | --- | --- | --- | --- | --- | --- |
|  | *P. punctifrons* | *P. poligraphus* | *P. subopacus* | *P. punctifrons* | *P. poligraphus* | *P. subopacus* |
| (*Z*)-DMCHE | 0 | 341 | 6808 | 0 | 6 (3, 13) | 13 (1, 122) |
| (*Z*)-DMCHA^a^ | 0 | 2 | 2 | 0 | 0 (0, 0) | 0 (0, 0) |
| (*E*)-DMCHA^a^ | 0 | 2 | 58 | 0 | 0 (0, 0) | 0 (0, 1) |
| Benzyl alcohol | 0 | 0 | 12 | 0 | 0 | 0 (0, 0) |
| (*Z*)-DMCHE  + (*Z*)-DMCHA^a^ | 0 | 226 | 5613 | 0 | 6 (3, 12) | 12 (1, 110) |
| (*Z*)-DMCHE  + (*E*)-DMCHA^a^ | 0 | 416 | 7924 | 0 | 5 (2, 11) | 17 (2, 150) |
| (*Z*)-DMCHE  + benzyl alcohol | 0 | 376 | 4741 | 0 | 8 (4, 17) | 11 (1, 101) |
| (*Z*)-DMCHE + racemic grandisol | 0 | 431 | 6748 | 0 | 6 (3, 13) | 16 (2, 141) |
| (*Z*)-DMCHE + (*E*)-DMCHE + (*Z*)-DMCHA + (*E*)-DMCHA + racemic grandisol | 0 | 226 | 4980 | 0 | 4 (2, 8) | 12 (1, 105) |
| Control (*n*-nonane) | 0 | 1 | 163 | 0 | 0 (0, 0) | 0 (0, 2) |

This study was conducted from 30 July until 8 September 2020. Traps were emptied and rotated twice per week. Estimated marginal means per trap per rotation (adjusted for random effects) are shown for each treatment, with a 95% confidence interval (95% CI). ^a^ The chemical purity of (*Z*)-DMCHA and (*E*)-DMCHA degraded quickly, thus our results for these compounds in 2020 were probably not reliable.

**2021.** *P. poligraphus* was attracted to traps baited with (*Z*)-DMCHE (*P* = 0.01), but the addition of other compounds to (*Z*)-DMCHE did not significantly alter the trap catches (Table S4). For the *P. subopacus* model, using a negative binomial error distribution resulted in

convergence problems. A Poisson model could be fit, but did not show a significant treatment effect. Since trap catches were very small, no conclusions could be drawn from this experiment. In the second field experiment (Table S5), *P. poligraphus* was attracted to (–)-(*R*)-terpinen-4-ol (*P* < 0.001) but the catches were not significantly affected when 2-phenyl ethanol was added to (–)-(*R*)-terpinen-4-ol.

**Table S4** Results from the field study in 2021, aimed at investigating the pheromone of *P. subopacus*

| Treatment | Total number of beetles | | | Mean per trap per rotation, (95% CI) | | |
| --- | --- | --- | --- | --- | --- | --- |
|  | *P. punctifrons* | *P. poligraphus* | *P. subopacus* | *P. punctifrons* | *P. poligraphus* | *P. subopacus* |
| (*Z*)-DMCHE | 0 | 273 | 251 | 0 | 4 (1, 15) | 0 (0, 2) |
| (*E*)-DMCHE | 0 | 10 | 2 | 0 | 0 (0, 1) | 0 (0, 0) |
| (*Z*)-DMCHA^a^ | 0 | 2 | 0 | 0 | 0 (0, 0) | 0 |
| (*E*)-DMCHA^a^ | 0 | 2 | 0 | 0 | 0 (0, 0) | 0 |
| (*Z*)-DMCHE  + (*E*)-DMCHE | 0 | 159 | 110 | 0 | 3 (1, 9) | 0 (0, 1) |
| (*Z*)-DMCHE  + (*Z*)-DMCHA^a^ | 0 | 112 | 131 | 0 | 4 (1, 12) | 0 (0, 1) |
| (*Z*)-DMCHE  + (*E*)-DMCHA^a^ | 0 | 97 | 86 | 0 | 3 (1, 9) | 0 (0, 1) |
| Control (*n*-nonane) | 0 | 0 | 0 | 0 | 0 | 0 |

This study was conducted from 22 July until 1 September 2021. Traps were emptied and rotated twice per week. Estimated marginal means per trap per rotation (adjusted for random effects) are shown for each treatment, with a 95% confidence interval (95% CI). ^a^A stabilizer, BHT, was added to (*Z*)-DMCHA and (*E*)-DMCHA to improve the chemical stability of these compounds during storage and in the field.

**Table S5** Results from the field study in 2021, aimed at investigating the pheromone of *P. poligraphus*

| Treatment | Total number of beetles | | | Mean per trap per rotation, (95% CI) | | |
| --- | --- | --- | --- | --- | --- | --- |
|  | *P. punctifrons* | *P. poligraphus* | *P. subopacus* | *P. punctifrons* | *P. poligraphus* | *P. subopacus* |
| (–)-Terpinen-4-ol (99% ee) | 0 | 2049 | 0 | 0 | 59 (13, 264) | 0 |
| 2-Phenyl ethanol | 0 | 1 | 0 | 0 | 0 (0, 0) | 0 |
| (–)-Terpinen-4-ol (99% ee)  + 2-phenyl ethanol | 0 | 2514 | 0 | 0 | 67 (15, 301) | 0 |
| Control (*n*-nonane) | 0 | 5 | 0 | 0 | 0 | 0 |

This study was conducted from 22 July until 11 August 2021. Traps were emptied and rotated twice per week. Estimated marginal means per trap per rotation (adjusted for random effects) are shown for each treatment, with a 95% confidence interval (95% CI).
